# Deficient Reporting and Interpretation of Non-Inferiority Randomized Clinical Trials in HIV Patients: A Systematic Review

**DOI:** 10.1371/journal.pone.0063272

**Published:** 2013-05-03

**Authors:** Adrian V. Hernandez, Vinay Pasupuleti, Abhishek Deshpande, Priyaleela Thota, Jaime A. Collins, Jose E. Vidal

**Affiliations:** 1 Health Outcomes and Clinical Epidemiology Section, Department of Quantitative Health Sciences, Lerner Research Institute, Cleveland Clinic, Cleveland, Ohio, United States of America; 2 Department of Medicine, Case Western Reserve University, Cleveland, Ohio, United States of America; 3 Postgraduate School, Universidad Peruana de Ciencias Aplicadas (UPC), Lima, Peru; 4 HIV/AIDS Unit, Department of Internal Medicine, Guillermo Almenara General Hospital, EsSalud, Lima, Peru; 5 Department of Infectious Diseases, Emilio Ribas Institute of Infectious Diseases, São Paulo, Brazil; University of Cape Town, South Africa

## Abstract

**Objectives:**

Non-inferiority (NI) randomized clinical trials (RCTs) commonly evaluate efficacy of new antiretroviral (ARV) drugs in human immunodeficiency virus (HIV) patients. Their reporting and interpretation have not been systematically evaluated. We evaluated the reporting of NI RCTs in HIV patients according to the CONSORT statement and assessed the degree of misinterpretation of RCTs when NI was inconclusive or not established.

**Design:**

Systematic review.

**Methods:**

PubMed, Web of Science, and Scopus were reviewed until December 2011. Selection and extraction was performed independently by three reviewers.

**Results:**

Of the 42 RCTs (n = 21,919; range 41–3,316) selected, 23 were in ARV-naïve and 19 in ARV-experienced patients. Twenty-seven (64%) RCTs provided information about prior RCTs of the active comparator, and 37 (88%) used 2-sided CIs. Two thirds of trials used a NI margin between 10 and 12%, although only 12 explained the method to determine it. Blinding was used in 9 studies only. The main conclusion was based on both intention-to-treat (ITT) and per protocol (PP) analyses in 5 trials, on PP analysis only in 4 studies, and on ITT only in 31 studies. Eleven of 16 studies with NI inconclusive or not established highlighted NI or equivalence, and distracted readers with positive secondary results.

**Conclusions:**

There is poor reporting and interpretation of NI RCTs performed in HIV patients. Maximizing the reporting of the method of NI margin determination, use of blinding and both ITT and PP analyses, and interpreting negative NI according to actual primary findings will improve the understanding of results and their translation into clinical practice.

## Introduction

Non-inferiority (NI) randomized controlled trials (RCT) are standard research methodology to demonstrate that a new experimental treatment is not worse than reference therapy (active comparator) in terms of efficacy. Human immunodeficiency virus (HIV) NI trials have emerged as the new standard design for HIV drug development in both antiretroviral (ARV)-naïve and –experienced patients [1]. Although increased efficacy rates of highly active antiretroviral therapies (HAART) have reduced space for newer antiretroviral agents with better efficacies [2], there is need for treatment simplification and newer alternative agents. This has led to a growing number of HIV NI trials in recent years.

The extended Consolidated Standards of Reporting Trials (CONSORT) statement of 2006 [3] has updated recommendations to guide the conduct and reporting of NI trials. Reports indicate the endorsement of CONSORT statement by journals is associated with significant improvement in the quality of reporting of RCTs [4]. However, there have been emerging concerns regarding deficiencies in adherence to guidelines and recommendations in design, statistical analysis and reporting of RCTs investigating NI [5], [6]. An important aspect in designing a NI trial is the need to provide a rigorous scientific justification for the choice of NI margin. There is a considerable risk of accepting less than effective experimental therapies from NI trials with non-rigorous margins. Other basic requirements of a well-designed NI trial would be sample size calculation taking NI margin into account, a clear description of the use of 1- or 2-sided confidence intervals and both per protocol (PP) and intention-to-treat (ITT) analysis [7].

Another area of concern is the reporting and interpretation of NI RCTs in trials wherein the NI was not established or was inconclusive. Investigators’ personal agendas such as personal, financial, and intellectual conflicts of interest can influence how research findings are presented. Authors can shape the way readers interpret their results in a variety of ways. Distorted presentation or interpretation of non-significant trials either consciously or unconsciously is known as “spin” [8].

Against this background, we performed a systematic review of the literature to identify NI RCTs involving antiretroviral therapies in ARV-naïve and –experienced patients and evaluated the methodological quality and reporting standards by applying the extended CONSORT statement for those trials. We also aimed to identify the strategies, extent and level of spin in trials in which NI was inconclusive or was not established.

## Methods

### Study Selection

A comprehensive literature search using PubMed-Medline from 1960 through December 31, 2011, EMBASE from 1980 through December 31, 2011, The Web of Science from 1980 through December 31, 2011, and Scopus from 1960 through December 31, 2011 was conducted. The following keywords were used: non-inferiority; clinical trial; trial; antiretroviral, highly active antiretroviral therapy; and HAART. The search strategy of PubMed is available in the Supporting Information Text S1.

We searched for NI RCTs published in any language. RCTs were defined as prospective trials evaluating healthcare interventions in participants randomly assigned to study groups. Non-inferiority trials was defined as RCTs which aim to demonstrate that the new intervention is not worse than the comparator by more than a specified small amount, the NI margin (delta margin).

All published studies that assessed the efficacy of new ARV drug combinations or new interventions in comparison to a standard therapy or intervention in both ARV-naïve and ARV-experienced HIV patients were included. We excluded articles that were not RCTs or were reviews and/or comments. Equivalence trials were excluded. Equivalence trials are trials that aim to demonstrate that the study and control treatment effects differ by no more than a specific amount, the equivalence margin.

A list of retrieved articles was reviewed independently by 3 investigators (AVH, VP, AD) in order to choose potentially relevant articles, and disagreements about particular studies were discussed and resolved.

### Data Extraction

Data extraction from selected studies was performed independently by 3 investigators (AVH, VP, AD). Disagreement was resolved by consensus. Using a standardized data extraction form, we collected information on lead author, study name, year of study or publication year, study sponsor, study location, duration of study, study design, study sample size, new drug arm, standard drug arm, primary outcomes and secondary outcomes.

### Evaluation of Methodological Quality

The following data was extracted from all selected studies: 1) Choice of NI margin, 2) Method of selection of NI margin, 3) Sample size calculation used NI margin, 4) 1- or 2-sided confidence intervals, 5) Blinding method, 6) Statistical analysis – PP, ITT or both– and 7) Main conclusion based on PP, ITT, or both. The establishment of NI was based on confidence intervals reported by investigators: when efficacy was measured by success rates the lower CI should be above the negative NI margin; when efficacy was measured by failure rates the upper CI should be below the positive NI margin. Other conclusions such as the not establishment of NI or inconclusive results followed the explanations of the CONSORT guidelines [3].

### Definitions and Evaluation of Spin

Spin was defined in the context of NI trials in which NI was not established or was inconclusive. Evidence of spin was ascertained when one or two of the following were present. 1) Highlighting NI when NI is not established or inconclusive or unclear. 2) Distracting the reader with other results (e.g. secondary outcomes or information from other studies) when NI is not established/inconclusive or is unclear.

Strategy of spin employed by the authors was determined. The strategies of spin considered were: 1) Focus on statistically significant results (within-group comparisons, secondary outcomes, subgroup analyses, modified population of analyses); 2) Interpreting the negative result of the primary outcome (i.e. not establishing NI or was inconclusive) as showing equivalence; and 3) Claiming or emphasizing NI despite not establishing NI or when inconclusive.

The extent of spin was assessed in the abstract: results section only, conclusions section only, or both. Extent of spin was also assessed in the main text: one section other than conclusions section (results section, or synthesis of the results in the discussion section), in the conclusions section only, in 2 sections, or in all 3 sections.

Lastly, the level of spin in conclusions of the abstract and conclusions of the main text was evaluated. High spin involved no uncertainty in the framing, no recommendations for further trials, and no acknowledgement of not establishing NI for the primary outcome; also, when the new treatment is recommended for use in clinical practice. Moderate spin involved some uncertainty in the framing or recommendations for further trials, but no acknowledgement of not establishing NI for the primary outcome. Low spin involved uncertainty in the framing and recommendations for further trials or acknowledgement of not establishing NI.

### Statistical Analysis

Primarily, we stratified the studies on the basis of history of ARV therapy: ARV-naïve studies *vs* ARV-experienced studies. The baseline risk of patients is different between the two groups: ARV-experienced patients are more likely to develop virological failure and resistance to ARV drugs compared to ARV-naïve HIV patients. Secondarily, we stratified studies by a) year published: before 2007 *vs* since 2007; studies have shown improvement in reporting standards in RCTs post-CONSORT [9] and b) type of sponsor: government *vs* pharmaceutical companies; studies have shown that industry sponsored trials tend to draw pro industry conclusions (sponsorship bias) [10]. We did not attempt to formally use p values for comparisons between ARV-naïve and ARV-experienced studies, as we anticipated a small sample in each group.

### Role of the Funding Source

This work was funded by the Department of Quantitative Health Sciences, Cleveland Clinic. The funders had no role in study design; data collection, analysis, or interpretation; in writing the report, or in the decision to submit the article for publication. The researchers are all independent from the funding source.

## Results

### Study Characteristics

**Figure 1:**
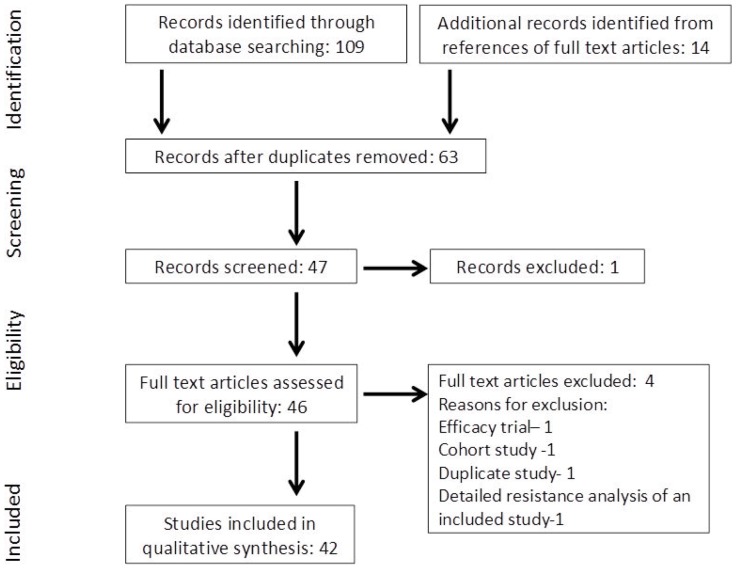
Search strategy profile of the systematic review.

Of the 109 citations retrieved and screened, 63 articles were identified and assessed for eligibility (Figure 1). Forty-two NI trials in HIV patients were selected from criteria described above and Table 1 and Table 2 summarize the main characteristics of trials in ARV-naïve and ARV-experienced patients, respectively. Of the 42 RCTs (n = 21,919; range 41–3,316) selected, 23 were in ARV-naïve [11]–[33] and 19 in ARV-experienced [34]–[52] patients. The earliest NI trial was published in the year 2000. The funding source for majority of the studies was pharmaceutical companies (alone or with a nonprofit source); 19 (83%) and 11 (58%) studies in ARV-naïve and ARV-experienced HIV patients, respectively. Government funding was the next most common source of funding; 4 (17%) and 7 (37%) studies in ARV-naïve and ARV-experienced HIV patients, respectively. Duration of the trials ranged from 16 weeks to 4.9 years. Primary outcomes were clearly identified in all NI trials, and the most common primary endpoint was the proportion of patients with HIV RNA levels <50 copies/mL.

**Table 1:** Study characteristics of non-inferiority trials in ARV-naive patients.

First Author/Year Published/Study Name	Sponsor	Country(ies)	Duration	New drug arm (n)	Standard arm (n)	Primary outcome(s)
Gallant/2004/Gilead 903 [11]	Pharmaceutical company	South America, Europe, USA	48 weeks	TDF 300/plac qd +3TC 150 bid+EFV600 qd (n = 299)	d4T40/plac bid +3TC150 bid+EFV600 qd (n = 301)	Proportion of patients with HIV RNA levels <400 copies/ml at week 48.
DeJesus/2004/CNA30024 [12]	Pharmaceutical company	USA, Europe, South America, Central America, Puerto Rico	48 weeks	ABC300/plac bid +3TC 150 bid+EFV200 qd (n = 324)	ZDV300/plac bid +3TC 150 bid+EFV200 qd (n = 325)	Proportion of subjects with plasma HIV-1 RNA levels ≤50 copies/ml at week 48 adjusted for randomization strata (HIV RNA level ≤100000 vs. >10000).
Gathe/2004/SOLO [13]	Pharmaceutical company	North America, Europe, South Africa, Australia	48 weeks	FPV/RTV 700×2/100×2+ ABC/3TCqd (n = 322)	NFV250×5 bid+ABC/3TC qd (n = 327)	Proportion of patients with vRNA <400 copies/ml at week 48.
Vibhagool/2004/CNA3014 [14]	Pharmaceutical company	Argentina, Brazil, Canada, Italy, Mexico, Thailand	48 weeks	ABC300+3TC 150/ZDV300 combination tab (COM) bid (n = 169)	IND800 tid/COM bid (n = 173)	Reduction in plasma HIV-1 RNA <400 copies/ml at week 48.
Moyle/2005/ZODIAC (CNA30021) [15]	Pharmaceutical company	USA, Canada, Spain, Brazil, Denmark, Germany, Poland, Argentina, Mexico, UK	48 weeks	ABC300×2/placx2qd +3TC150×2 qd+EFV200×3 qd (n = 392)	ABC300/plac bid +3TC 150×2 qd+EFV200×3 qd (n = 392)	Proportion of patients with plasma HIV-1 RNA <50 copies/ml at week 48 adjusted for randomized strata (HIV RNA level ≤100000 vs >100000).
Eron/2006/KLEAN [16]	Pharmaceutical company	USA, Europe, Canada	48 weeks	FPV-RTV (700/100 bid)+ABC-3TC (600/300 qd) (n = 434)	LPV-RTV(400/100 bid)+ABC-3TC (600/300 bid) (n = 444)	Proportion of patients with HIV-1 RNA concentration <400 copies/ml at 48 weeks and the proportion who permanently discontinued randomized treatment due to adverse events.
Johnson/2006/M02–418 [17]	Pharmaceutical company	7countries (North America, Asia, Europe, Australia)	48 weeks	LPV800/RTV200 qd+TDF300 qd+FTC200 qd (n = 120)	LPV400/RTV100 bid+TDF300 qd+FTC200 qd (n = 80)	Proportion of subjects with plasma HIV-1 RNA ≤50 copies/ml at week 48.
Pozniak/2006/Study 934 [18]	Unclear	France, Germany, Italy, Spain, UK, USA	48 weeks	EFV600+ TDF300+ FTC200 qd (n = 258)	EFV600 qd+ZDV300/3TC150 bid (n = 259)	Proportion of patients with HIV RNA levels <400 copies/ml through week 48 according to the FDA algorithm for the TLOVR.
Molina/2008/CASTLE [19]	Pharmaceutical company	29 countries	48 weeks	ATV-RTV (300/100 qd)+TDF-FTC (300/200 qd) (n = 440)	LPV-RTV(400/100 bid)+TDF-FTC(300/200 qd) (n = 443)	Proportion of patients with HIV RNA levels of <50 copies/ml at 48 weeks.
Ortiz/2008/ARTEMIS [20]	Pharmaceutical company	26 countries	48 weeks	DRV-RTV(800/100) qd+TDF-FTC(300/200 qd)(n = 343)	LPV-RTV(800/200 [bid or qd])+TDF-FTC(300/200 qd) (n = 346)	HIV-1 RNA <50 copies/ml PP time to loss of virological response at 48 weeks.
Rey/2009/DAUFIN [21]	Pharmaceutical company	France	48 weeks (study planned for 96 weeks )	TDF(245)/3TC/NVP(300/400) qd (n = 36)	ZDV/3TC(300/150)/NVP(200) bid (n = 35)	Proportion of patients with HIV RNA <400 copies/ml at all visits through week 96.
Kumar/2009/ACTION [22]	Pharmaceutical company	USA, Mexico	48 weeks	ABC/3TC/ZDV bid (n = 139)	ATV 2×200 qd +3TC/ZDV bid (n = 140)	Proportion of patients with HIV-1 RNA <50 copies/ml at week 48 who did not meet the definition of virologic failure through week 48 using an intent-to-treat exposed analysis; frequency of treatment-limiting adverse events, grade 2–4 adverse events and serious adverse events over week 24 and 48 weeks.
Lennox/2009/STARTMRK [23]	Pharmaceutical company	14 countries	48 weeks	RAL based combination regimen (400 bid)+TDF/FTC (n = 281)	EFV based combination regimen (600 qd)+TDF/FTC (n = 282)	Achievement of <50 vRNA copies/ml at week 48.
Mugyenyi/2010/DART [24]	Government; Pharmaceutical company	Uganda, Zimbabwe	4.9 years	clinically driven monitoring of HIV antiretroviral therapy (CDM group) (n = 1660)	laboratory (CD4 for efficacy and hematology and biochemistry for safety) and clinical monitoring of antiretroviral therapy (LCM group) (n = 1656)	Progression to a new WHO stage 4 HIV event or death; serious adverse events, which are defined as events not related only to HIV and either fatal, life threatening, causing unplanned or prolonged admission to hospital, causing permanent or significant disability, or other important medical conditions.
Molina/2010/CASTLE [25]	Pharmaceutical company	29 countries	96 weeks	ATV-RTV(300/100 qd)+TDF-FTC(300/200 qd) (n = 440)	LPV-RTV (400/100 bid)+TDF-FTC(300/200 qd) (n = 443)	Proportion of patients with HIV RNA <50 copies/ml at week 48.
Sierra-Madero/2010/NCT00162643 [26]	Government	Mexico	48 weeks	EFV 600 qd+AZT/3TC 300/150 bid (n = 95)	LPV/RTV 400/100 bid+AZT/3TC 300/150 bid (n = 94)	Proportion of patients with HIV-1 RNA <50 copies/ml at week 48.
Soriano/2011/ARTEN [27]	Pharmaceutical company	Argentina, Germany, Italy, Mexico, Poland, Portugal, Romania, Spain, UK, Switzerland	48 weeks	NVP200 bid+fixed dose TDF300/FTC200 qd; NVP 400 qd+fixed dose TDF300/FTC200 qd (n = 376)	ATV300+ RTV100 qd+fixed dose TDF300/FTC200 qd (n = 193)	Confirmed plasma HIV RNA <50 copies/ml at week 48 using the TLOVR algorithm which defines treatment response as plasma HIV RNA <50 copies/ml at two consecutive weeks upto week 48.
Cohen/2011/THRIVE [28]	Pharmaceutical company	21 countries	48 weeks	RPV25 qd+N(t)RTIs (n = 340)	EFV600 qd+N(t)RTIs (n = 340)	Percentage of all patients who received at least one dose of RPV or EFV who had a confirmed virological response (viral load <50 copies/ml) at 48 weeks.
Firnhaber/2011/NCT00100646 [29]	Government	South Africa	72 weeks	sequential 2,4 and 8 week ART interruptions (n = 27)	continuous ART (n = 26)	Proportion of CD4+ T cell count measurements >350 cells/µl over 72 weeks of follow up following randomization.
Gathe/2011/NCT00561925 [30]	Pharmaceutical company	North America, Australia, Latin America, Africa, Europe	48 weeks	NVP XR400 qd (plus placebo)+TDF300+ FTC200 qd (n = 505)	NVP IR200 qd (plus placebo)+TDF300+ FTC200 qd (n = 506)	Sustained virologic response through week 48 (viral load <50 copies/ml).
Laurent/2011/ESTHER (ANRS12110) [31]	Government	Cameroon	2 years	clinical monitoring alone (n = 256)	clinical and lab monitoring (n = 237)	Mean increase in CD4 cell count from treatment initiation to month 24.
Molina/2011/ECHO [32]	Pharmaceutical company	USA, Canada, Australia, South Africa, 10 countries in Europe, 3 in Asia, and 4 in Latin America	48 weeks	RPV25 qd+TDF+FTC (n = 346)	EFV600 qd+TDF+FTC (n = 344)	Percentage of patients with confirmed response at week 48.
Eron/2011/MDRK [33]	Pharmaceutical company	6 continents	48 weeks	RAL800 qd+TDF-FTC(300/200) qd (n = 386)	RAL400 bid+TDF-FTC(300/200) qd (n = 389)	Virological response at 48 weeks (vRNA loads <50 copies/ml) in patients who received at least one dose of study drug.

Plac = placebo; TLOVR = time to loss of virologic response; TDF = Tenofovir DF; 3TC = Lamivudine; EFV = Efavirenz; d4T = Stavudine; ABC = Abacavir; ZDV = Zidovudine; FPV = Fosamprenavir; RTV = Ritonavir; NFV = Nelfinavir; IND = Indinavir; FTC = Emtricitabine; ATV = Atazanavir; DRV = Darunavir; NVP = Nevirapine; RAL = Raltegravir; AZT = Azidothymidine; RPV = Rilpivirine.

**Table 2:** Study characteristics of non-inferiority trials in ARV-experienced patients.

First Author/Year Published/Study Name	Sponsor	Country(ies)	Duration	New drug arm (n)	Standard arm (n)	Primary outcome(s)
Eron/2000 [34]	Unclear	USA, Puerto Rico	16 weeks	Combivir (3TC150/ZDV300) bid+currently prescribed PI (n = 110)	3TC150 bid+ZDV200 tid+a FDA-approved PI (n = 113)	Treatment failure defined as HIV-1 RNA ≥0.5log10 above baseline in patients with viral load>LLOQ (400 copies/ml) and as HIV-1 RNA increasing to ≥1250 copies/ml in patients with viral load<LLOQ at randomization.
Nadler/2003/STARR (ESS40011)* [35]	Pharmaceutical company	USA	24 weeks	APV600/RTV bid+≥2 non-PIs (n = 158)	APV1200 bid+≥2 non-PIs (n = 53)	Proportion of patients with plasma HIV-1 RNA levels <200 copies/ml at week 24.
Benson/2004/FTC-303/350 [36]	Pharmaceutical company	USA	48 weeks	FTC200 qd+d4T or ZDV+a PI or NNRTI (n = 294)	3TC150 bid+d4T or ZDV+a PI or NNRTI (n = 146)	Virologic failure at week 48 defined as two consecutive measurements of HIV-1 RNA >400 copies/ml.
Madruga/2007/TITAN [37]	Pharmaceutical company	26 countries	48 weeks	optimized background regimen+DRV-RTV600/100 bid (n = 298)	optimized background regimen+LPV-RTV400/100 bid (n = 297)	Proportion of patients with confirmed HIV-1 RNA <400 copies/ml in plasma at week 48.
Marchou/2007/ANRS 106 [38]	Government	France	96 weeks	Fixed intermittent treatment: 8 weeks off therapy followed by 8 weeks on therapy (n = 200)	Constant combination ART (n = 203)	Cumulative proportion of patients throughout the study reaching a confirmed CD4 cell count of less than 300×10^6^ cells/L, defined as immunological failure.
Pulido/2008/NCT00114933 [39]	Pharmaceutical company	Spain	48 weeks	LPV/RTV monotherapy as maintenance therapy (n = 103)	LPV/RTV +2 NRTIs as maintenance therapy (n = 102)	Proportion of patients without therapeutic failure at 48 weeks.
De Castro/2009/EASIER [40]	Government	France	24 weeks	Switch to RAL (400 bid)+backgroundregimen (n = 85)	T-20(90 subcutaneous bid)+background regimen (n = 85)	Proportion of patients with virologic failure defined as confirmed plasma HIV-1 RNA level of ≥400 copies/ml during the 24 weeks of study.
Porter/2009/ICARUS [41]	Government	USA	6 months	IL-2 alone as maintenance therapy (n = 27)	IL-2 and HAART as maintenance therapy (n = 14)	CD4 T cell count at month 6 (and at least 4 weeks after last IL-2 cycle), with treatment success defined as maintaining randomization assignment and having a CD4 T cell count at least 90% of baseline.
Girard/2009/COOL [42]	Pharmaceutical company	France	48 weeks	qd maintenance regimen of a two-drug combination - TDF300 and EFV600 (n = 71)	Conventional qd three drug combination - TDF300, 3TC300 and EFV600 (n = 72)	Proportion of patients with plasma HIV-1 RNA <50 copies/ml at week 48 in the absence of treatment modification.
Martinez/2010/SPIRAL [43]	Government; Pharmaceutical company	Spain	48 weeks	Switch to RAL-based therapy from RTV-boosted PI (n = 139)	RTV-boosted protease inhibitor-based therapy (n = 134)	Proportion of patients who were free of treatment failure at 48 weeks.
Katlama/2010/MONOI-ANRS 136 [44]	Government	France	48 weeks	DRV/RTV monotherapy (stop the two NRTIs) (n = 112)	Continuation of triple drug DRV/RTV-containing regimen (n = 113)	Proportion of patients with treatment success (HIV RNA <400 copies/ml) by week 48.
Arribas/2010/MONET [45]	Pharmaceutical company	11 European countries, Russia, Israel	48 weeks	DRV/RTV 800/100 qd (monotherapy arm) (n = 127)	DVR/RTV 800/100 qd+two nucleoside analogues (triple therapy arm) (n = 129)	Treatment failure defined as two consecutive HIV RNA levels >50 copies/ml at week 48 or discontinuation of randomized treatment.
Zajdenverg/2010/M06–802 [46]	Pharmaceutical company	17 countries in North America, South America, Europe, Africa, Australia	48 weeks	LPV/RTV 800/200 qd +2 NRTIs.(n = 300)	LPV/RTV 400/100 bid +2 NRTIs (n = 299)	Proportion of patients responding with HIV-1 RNA <50 copies/ml at week 48.
Meynard/2010/KALESOLO [47]	Government	France	48 weeks	LPV/RTV monotherapy (400/100 bid) (n = 87)	Current combined antiretroviral treatment (cART) (n = 99)	Proportion of patients with viral load <50 copies at week 48 without modification of antiretroviral treatment during the study.
Sanne/2010/NCT00255840 [48]	Government	South Africa	96 weeks	nurse management of doctor-initiated ART care (n = 404)	doctor management of doctor-initiated ART care (n = 408)	A composite endpoint of possible treatment-limiting events that could occur on first-line ART. These outcomes were: all-cause mortality, loss to follow-up, virologic failure, toxicity failure, withdrawn consent, defaulting clinic schedule, and HIV-disease progression.
Reynolds/2010/NCT00339456 [49]	Government	Uganda	72 weeks	5 days on, 2 days off ART/7 days on 7 days off ART (n = 57/32)	continuous ART (n = 57)	ART treatment failure determined by a plasma HIV RNA ≥10,000 copies on any one evaluation, a plasma HIV RNA level ≥1,000 copies on two consecutive measurements, a plasma HIV RNA level >400 copies/ml at the end of the study, a CD4+ cell count decrease of >30% from baseline on 2 consecutive measurements, death attributed to study participation or occurrence of an opportunistic infection.
Campo/2010/NCT0013745382 [50]	Pharmaceutical company	USA	48 weeks	(EFV-A): qd EFV(600), qd 3TC(300) and qd enteric-coated ddI (400 or 250 if weight <60 kg) (n = 131)	(EFV-B): qd EFV(600) plus continuation of current NRTIs (n = 131)	Proportion of patients who maintained plasma HIV-1 RNA levels <50 copies/ml at week 48.
Eron/2010/SWITCHMRK 1 AND 2 [51]	Pharmaceutical company	5 continents	24 weeks	RAL-based regimen (n = 350)	LPV-RTV-based regimen (n = 352)	Mean % change in lipid concentrations from baseline to week 12, the proportion of patients with vRNA concentration <50 copies/ml at week 24, and the frequency of adverse events up to 24 weeks.
Clumeck/2011/MONET [52]	Pharmaceutical company	Russia, Israel, 11 European countries	96 weeks	DRV/RTV800/100 qd (monotherapy arm) (n = 129)	DRV/RTV800/100 qd+two nucleoside analogs (triple therapy arm) (n = 127)	Treatment failure defined as two consecutive HIV RNA levels >50 copies/ml at week 96 or discontinuation of randomized treatment.

* 81% ARV-experienced patients in the study; LLOQ = Lower limit of quantitation; 3TC = Lamivudine; ZDV = Zidovudine; APV = Amprenavir; RTV = Ritonavir; FTC = Emtricitabine; d4T = Stavudine; DRV = Darunavir; LPV = Lopinavir; RAL = Raltegravir; T-20 =  Enfuvirtide; TDF = Tenofovir DF; EFV = Efavirenz; ddI = Didanosine.

### Assessment of Methodological Quality

All 42 RCTs assessed were of parallel design. Four trials [11], [14], [34], [36] were reported as equivalence trials when in fact they were NI trials. Majority of the studies (38/42) explained why a NI trial was performed as opposed to superiority or equivalence trials. Thirteen trials were phase 3, one was phase 2, one was phase 4, and 27 studies did not clearly state the trial phase. Twenty-seven trials (64%) reported the similarity of the standard arm (comparative arm) to previous efficacy trials respect to inclusion/exclusion criteria, types of drugs and outcomes. None of the studies were placebo-controlled.

The study design characteristics stratified by ARV-naïve and ARV-experienced trials are summarized in Table 3. All studies identified a pre-specified NI margin. All but two of the studies described the NI margin between 7% and 25%. In one ARV-naïve [24] study and one ARV-experienced [48] study upper 95% CI limit of the Hazard ratio was the NI margin. The recommended NI margin between 10 and 12% [53], [54] was used in 17/23 (74%) ARV-naïve and 11/19 (58%) ARV-experienced studies. Only 9 (39%) studies in ARV-naïve group and 3 (16%) studies in the ARV-experienced group reported justification for their choice of NI margin or limits of Hazard ratios. In 2 studies, NI margin was selected based on investigators assumptions [12], [24]; 4 studies based on other publications or reviews [11], [14], [35], [46]; 2 studies based on guidelines [28], [32]; 2 studies, calculated based on previous trial results [20], [31]; one study based on investigators assumption and other publications and reviews [15]; one study based on guidelines and calculated from previous trial results [37]. Sample size calculation used the NI margin in 8 (35%) and 13 (68%) studies in ARV-naïve and ARV-experienced trials, respectively. A double-blind design approach was employed in 8 ARV-naïve trials and one ARV-experienced trial. All trials reported results using the confidence intervals approach; 2-sided confidence intervals were used in 22 (96%) and 15 (79%) studies in ARV-naïve and ARV-experienced trials, respectively. Although both ITT and PP analysis were performed in 10 (44%) and 9 (47%) trials in ARV-naïve and ARV-experienced trials respectively, only in 5 studies in the ARV-experienced group was the main conclusion based on both analyses (Table 4). Two studies each in ARV-naïve and ARV-experienced trials gave their main conclusion based on PP analyses, the main analysis for NI trials (Table 4). The type of statistical analysis performed was not clear in 2 of the trials in the ARV-naïve group. Only use of NI margin to calculate sample size and blinding method used were significantly different between the two groups of studies (Table 3).

**Table 3:** Study design characteristics stratified by type of trial population.

	Trials in ARV-naïve patients (n = 23)	Trials in ARV-experienced patients (n = 19)
NI margin (%)		7 (1)
	10 (6)	10 (3)
	12 (11)	12 (8)
	13 (1)	12.5 (1)
	15 (3)	14 (1)
	25 (1)	15 (3)
	NA* (1)	20 (1)
		NA** (1)
Method of selection of NI margin	Guidelines (2)	
	Investigator’s assumption (2)	
	Other publications or reviews (2)	Other publications or reviews (2)
	Calculated by investigator based on previous trials’results (2)	Guidelines and calculated by investigator based on previous trials’ results (1)
	Investigator’s assumption and other publications orreviews (1)	Not clear (16)
	Not clear (14)	
Sample size calculation used NI margin	No (15)	No (6)
	Yes (8)	Yes (13)
1 or 2 sided confidence intervals	1-sided (1)	1-sided (4)
	2-sided (22)	2-sided (15)
Blinding method	Open label (15)	Open label (18)
	Double blind (8)	Double blind (1)
Statistical analysis	Intention-to-treat (10)	Intention-to-treat (9)
	Per protocol (1)	Per protocol (1)
	Intention-to-treat and per protocol (10)	Intention-to-treat and per protocol (9)
	Not clear (2)	
Main conclusion based on	Intention-to-treat (19)	Intention-to-treat (12)
	Per protocol (2)	Per protocol (2)
	Not clear(2)	Intention-to-treat and per protocol (5)

* Upper 95% confidence limit for Hazard ratio was no greater than 1.18; **Upper 95% confidence limit for the Hazard ratio was less than 1.40.

**Table 4:** Primary outcome results.

ART-naïve studies	Main result of primary outcome: new treatmentvs control arm	ART-experienced studies	Main result of primary outcome: new treatment vs control arm
Gallant *et al* 2004^#^	80% vs 84%; RD −4%, 95%CI −10.4, 1.5	Eron *et al* 2000*	96.4% vs 92.9%; RD 3.5%, lower 95%CI −2.4
DeJesus *et al* 2004*	70% vs 69%; RD 1%, 95%CI −6.3, 7.9	Nadler *et al* 2003*	62% vs 53%; RD 9%, lower 95%CI −6
Gathe *et al* 2004*	69% vs 68%; RD 1%, 95%CI −6, 8	Benson *et al* 2004*	7% vs 8%; RD −0.6%, 95%CI −4.4, 3.1
Vibhagool *et al* 2004^$^	66% vs 50%; RD 16.6%, 95%CI 6.0, 27.2	Madruga *et al* 2007^$^	77% vs 68%; PP: RD 9%, 95%CI 2, 16
Moyle *et al* 2004*	66% vs 68%; RD −1.7%, 95%CI −8.4, 4.9	Marchou *et al* 2007*	3.6% vs 1.5%; RD: 2.1%, upper 95%CI 5.6
Eron *et al* 2006*	73% vs 71%; RD 2%, 95%CI −4.8, 7.1	Pulido *et al* 2008*	94% vs 90%; PP: RD 4%, 95%CI 3.4,11.8
Johnson *et al* 2006*	70% vs 64%; RD 6%, 95%CI 7, 20	De Castro *et al* 2009*	1.2% vs 1.2%; PP: RD 1.22%, 95%CI −5.6, 8.1; ITT: RD 0.01%, 95%CI −6.7, 6.8
Pozniak *et al* 2006^$^	84% vs 73%; RD 11%, 95%CI 4, 19	Porter *et al* 2009^##^	48.1% vs 92.3%; RD −44.2%, 95%CI −64.2, −11.2
Molina *et al* 2008*	78% vs 76%; RD 1.7%, 95%CI −3.8, 7.1	Girard *et al* 2009^#^	90% vs 100%; PP: RD −10%, lower 95%CI −16.4; and 81.7% vs 97.2%; ITT: RD −15.5%, lower 95%CI −23.7
Ortiz *et al* 2008*	84% vs 78%; PP: RD 5.6%, 95%CI −0.1, 11.0	Martinez *et al* 2010*	89.2% vs 86.6%; RD 2.6%, 95%CI −5.2, 10.6
Rey *et al* 2009^%^	NR	Katlama *et al* 2010^$$^	94% vs 99%; PP: RD −4.9%, 90%CI −9.1, −0.8; and 87.5% vs 92%; ITT: RD −4.5%, 90%CI −11.2, 2.1
Kumar *et al* 2009*	62% vs 559%; RD 3%, 95%CI −5.9; 10.4	Arribas *et al* 2010*	86.2% vs 87.8%; PP: RD −1.6%, 95%CI −10.1, 6.8; and 84.3% vs 85.3%; ITT: RD −1.0%, 95%CI −9.9, 8.8
Lennox *et al* 2009*	86% vs 82%; PP: RD 4.2, 95%CI −1.9, 10.3	Zajdenverg *et al* 2010*	55.3% vs 51.8%; RD 3.5%, 95%CI −4.5, 11.5
Mugyenyi *et al* 2010**	28% vs 21%; HR 1.31, 95%CI 1.14, 1.51	Meynard *et al* 2010^#^	84% vs 88%; RD −4.0%, 90%CI −12.4, 4.5
Molina *et al* 2010^$^	74% vs 68%; RD 6.1%, 95%CI 0.3, 12.0	Sanne *et al* 2010*	48% vs 44%; HR 1.09, 95%CI 0.89,1.33
Sierra-Madero *et al* ^$^ 2010	70% vs 53%; RD: 17%, 95%CI 3.5, 31	Reynolds *et al* 2010*	88.5% vs 78.4%; RD 10.1%, 97.5%CI −6, 26
Soriano *et al* 2011*	66.8% vs 65.3%; RD 1.9%, 95%CI −5.9, −9.8	Campo *et al* 2010*	81% vs 79%; RD 1.4%, lower 97.5%CI −8.4
Cohen *et al* 2011*	86% vs 82%; RD 3.5%, 95%CI 1.7, 8.8	Eron *et al* 2010^##^	Study 1: RD −6.6%, 95%CI −14.4, 1.2; Study 2: RD 5.8%, 95%CI −12.2, 0.2; 84.4% vs 90.6%; Combined: RD −6.2%; 95%CI −11.2, −1.3
Firnhaber *et al* 2011^#^	82.1% vs 93.7%; RD −11.95%, 97.5%CI −24.1, 0.2	Clumeck *et al* 2011^#^	78% vs 82%; PP: RD −4.2%, 95%CI −14.3, 5.8; 75% vs 81%; ITT: RD −5.8%, 95%CI −16.0, 4.4
Gathe *et al* 2011*	81.0% vs 75.9%; RD 4.9%, 95%CI −0.1, 10		
Laurent *et al* 2011^#^	175 cells/microL (SD 190, 95% CI 151–200) vs 206 cells/microL (SD 190, 95% CI 181–231) MD −31, upper 95%CI −45		
Molina *et al* 2011*	83% vs 83%; RD −0.4, 95%CI −5.9, 5.2		
Eron *et al* 2011^##^	83% vs 89%; RD −5.7%, 95%CI −10.7, −0.83		

RD = risk difference; MD = mean difference; HR = hazard ratio; PP = per protocol; ITT = intention-to-treat; NR = not reported; * = NI established; ^#^ = NI not established; ^$^ = superior; ^%^ = study terminated early; ** = study inconclusive; ^##^ = inferior; ^$$^ = NI established by PP analysis, NI not established by ITT analysis.

In ARV-naïve trials, NI was established in 13 (57%) studies (Table 4). Of these, one study [13] did not comment on the establishment of NI, although NI was established; NI was not established in 7 studies. Of these, one study [11] concludes “equivalence not established”, when NI is not established. In one study [14], the authors do not acknowledge that NI is not established and conclude “equivalence established” when in fact the result is superior. Two studies [25], [26] conclude NI established when the results are actually superior. One study [18] concludes exclusion of inferiority when the results are superior. Two trials though inconclusive are not reported so: one trial [33] is reported as inferior while it has discordant results with ITT and PP analysis, with superiority established by PP analysis and one trial [24] mentions “the upper (but not lower) CI was higher than the pre-defined margin of NI”. One study [21] was terminated early because of slow recruitment and high rate of early virological failures.

In ARV-experienced trials, NI was established in 12 (63%) studies (Table 4). Of these, one study [36] concluded “equivalence established” when in fact NI was established. NI was not established in 5 studies. Of these, one study [37] concludes NI established when the result is actually superior. One study [41] was inferior and was rightly acknowledged so by the authors. One trial [44] was inconclusive with authors mentioning appropriately that they cannot conclude NI as conclusions were discordant (NI established by PP analysis but not by ITT analysis) with respect to the NI margin.

Additional benefit with the new drug arm was claimed in 22 (96%) of the ARV-naïve trials (10 studies with NI not established/inconclusive/inferior), and in 11 (58%) of the ARV-experienced trials (6 studies with NI not established or inconclusive). Additional benefits most commonly claimed were less adverse events, improved lipid profile and low rates of virological failure. All studies claiming additional benefit clearly explained the benefits. All but two studies [21], [40] claiming additional benefit had analysis performed to support their claims.

Study design characteristics stratified by year of publication and type of sponsor are summarized in Table S1 and Table S2, respectively. None of the study design characteristics were different between the two groups when stratified by year published; only use of NI margin to calculate sample size and blinding method used had different distributions between the two groups of studies when stratified by type of sponsor.

### Assessment of Spin

Strategies, extent and level of spin employed by the 16 HIV NI trials (9 ARV-naïve trials [11], [14], [18], [24]–[26], [29], [31], [33], 7 ARV-experienced trials [37], [41], [42], [44], [47], [51], [52]) are shown in Table 5. Spin as per definition was assessed in NI trials wherein NI was not established or was inconclusive. NI was not established or was inconclusive in 9/23 (39%) of ARV-naïve trials and 7/19 (37%) of ARV-experienced trials. Of these, spin was identified in 7/9 (78%) and 4/7 (57%) studies in ARV-naïve [11], [14], [18], [24]–[26], [29] and ARV-experienced trials [37], [42], [47], [52], respectively.

**Table 5:** Spin in trials where non-inferiority was not established or was inconclusive by type of trial population.

Spin	Trials in ARV-naïve patients (n = 9)	Trials in ARV-experienced patients (n = 7)
Strategy of Spin		
Focus on statistically significant results (within-group comparisons, secondary outcomes, subgroup analysis, modified population of analysis)	4	4
Interpreting the negative results of primary outcome as showing equivalence	2	0
Claiming or emphasizing the non-inferiority despite not-establishing non- inferiority/inconclusive	5	1
Extent of spin in abstract		
Results section only	0	0
Conclusions section only	4	0
Results and conclusions sections	3	3
Level of spin in conclusions of the abstract		
High spin	5	1
Moderate spin	1	0
Low spin	1	2
Extent of spin in main text		
Discussion section only	1	1
Conclusions section only	0	1
Discussion and conclusions sections	2	1
Results and discussion sections	2	1
Results and conclusions sections	1	0
Results, discussion and conclusions sections	1	0
Level of spin in conclusions of the main text		
High spin	3	1
Moderate spin	3	0
Low spin	1	3

Of the 15 studies in which spin was identified, the most common (8/15) strategy of spin employed was focusing and highlighting of statistically significant results which included within-group comparisons, secondary outcomes, subgroup analyses, and/or modified population of analyses. In total, 10 abstracts were classified as having spin, of which 6 had spin in both results and conclusions sections and 4 in conclusions section only. Level of spin in conclusions section of abstract was ‘high’ in 6 studies. In total, 11 articles were classified as having spin in their main text. More than 50% of the articles (8/15) had spin in at least two sections of the main text while 3 studies had spin in one section of the main text. Level of spin in conclusions section of the main text was ‘high’ in 4 studies.

Strategies, extent and level of spin in studies stratified by year published and type of sponsor are summarized in Table S3 and Table S4, respectively. No differences were observed.

## Discussion

### Principal Findings

We investigated the methodological quality and reporting standards of RCTs of HIV NI trials. The overall quality of HIV NI RCTs was poor. The main deficiencies were lack of reference of historical data on the active comparator, no information on method of selection of NI margin, not taking the NI margin into account while determining sample size, inadequate blinding of patients, and failure to perform both ITT and PP analysis. Other flaws encountered less frequently were usage of terms equivalence and NI interchangeably and not clearly stating so when the trial results are inconclusive or superior. We also identified high frequency of spin in NI trials in which NI was not established or was inconclusive. Most common strategy of spin observed in these trials was the focus on statistically significant results for other analyses.

### Methodological and Reporting Standards in HIV Literature

The findings of our study are consistent with previous studies assessing methodological quality and reporting in HIV NI trials. Parienti *et al* investigated methodological standards of NI HIV trials reported in pre-specified select journals of high impact factor between 2001 and 2006 [2]. Four out of 18 studies provided rationale for the NI margin and 7/18 studies performed only ITT analysis for their primary endpoint. In studies with both ITT and PP analysis, main conclusion was based on ITT analysis, with the exception of one study. In a review of company-sponsored phase 3 NI trials between 2000 and 2007, Hill *et al* discussed the implications of study design for the choice of endpoints and sample size calculations [54]. They report inconsistencies in design and interpretation of HIV NI trials and stress on the importance of adopting standardized guidelines in conducting NI trials. In a recent study, statistical methods of 11 HIV NI trials published in 2010 were analyzed [1]. They noted that the conclusions of these trials were heavily dependent on statistical methods used to estimate confidence intervals. Both two-sided 95% CI and the one-sided 97.5% CI can be used for assessment of NI. The clue is not to reach a wrong conclusion using the wrong CI or alpha level. There is also the case that both CIs can reach different conclusions, but this is an uncommon situation [55].

Statistical decision procedures based on confidence limits are not the only valid and efficient inferential methods for establishing NI. Kaul *et al* [55] also refer to the use of the hypothesis-testing framework. Here, the null hypothesis of inequality (risk difference is greater than or equal to the margin) is rejected in favor of the alternative hypothesis of equality (risk difference is less than the margin) if the 1-sided P value is less than 0.025. These authors concluded that the judgment of NI is based on 3 prerequisites: 1) The new treatment exhibits therapeutic NI to the standard treatment; 2) the new treatment would exhibit therapeutic efficacy in a placebo-controlled trial, if such a trial were performed; and 3) the new treatment offers ancillary benefits with respect to safety, tolerability, convenience, or cost. The establishment of therapeutic NI is based on the a priori definition of NI margin, the adequate power of the trial, the consistency of the active control effect with that in historic trials, the similarity of design and conduct with historic trials, and the stability of the NI with alternative analytical criteria (tighter NI margin, relative vs. absolute risk, 1-sided vs. 2-sided CI, and ITT vs. PP analysis). We have described and discussed several of the topics related to the establishment of therapeutic NI. None of our studies had placebo control arms; however, none of the trials analyzed whether the new treatment would exhibit therapeutic efficacy in a placebo-controlled trial, if such a trial were performed. Most of the studies described ancillary benefits as motivation for their design.

### Spin in RCTs

Assessing RCTs for flaws in reporting and interpretation in terms of strategies, extent and level of ‘spin’ is a relatively new concept. Boutron *et al* identified the nature and frequency of spin in superiority RCTs with statistically non-significant results for primary outcomes [8]. All RCTs published in the month of December 2006 were analyzed and 72/205 RCTs were found to have statistically nonsignificant results. The strategies of spin were diverse, 68% and 61% of the abstracts and the main text, respectively were found to have spin in at least 1 section with high level of spin in 33% of abstracts conclusions section and 26% of the main-text conclusions. We adopted the definitions and classification scheme of spin from this study and applied them in the context of HIV NI trials where NI was not demonstrated or inconclusive.

### What our Results Add to Existing Literature

Double blinding was only used in 9 trials, although guidelines suggest using blinding whenever possible to minimize the risk of bias, especially information bias [6]. Although all included NI HIV trials pre-specified the NI margin, most did not explain the reasoning behind the selection of a given NI margin, and most of ARV-naive trials did not use the NI margin for sample size calculations. In most of the studies, the reasoning provided for selection of NI margin was not scientifically well grounded and were based on investigators assumption or based on other publications or reviews. We could not determine whether this was due to space limitations or due to a real lack of definition in the trial protocol. The clinical and statistical reasoning behind the selection of the appropriate NI margin is essential to be appropriately described in the manuscript [2], [5], [6]. Two thirds of the included trials reported the similarity of the current standard arm to previous trials (where the efficacy of the standard arm was established) with respect to outcomes, drug doses and inclusion/exclusion criteria. Any differences in these items should be described and justified [3]. Also, most of trials based their study conclusion on ITT analysis only. NI trials favor the PP analysis, which excludes patients with major protocol violations; by excluding these patients, which is expected to make the groups more similar, it is thought that analysis of the PP population may be more likely to show differences between treatments. However, both ITT and PP analyses are required to demonstrate NI [6], [51], and this was only true in 5 of our selected trials. Also, reporting of most methodological characteristics was found to be lacking irrespective of the history of ARV therapy, year published and type of sponsor.

We have developed a methodology to evaluate the presence, extension and degree of spin in NI trials, following the recommendations of a non-significant superiority trial environment [8]. Among the trials where NI was not demonstrated or was inconclusive, it was quite common to deviate the attention of readers to significant secondary analyses, and also to conclude ‘equivalence’ or even to stress the finding of NI where there is no. We also found that studies with spin showed it in several sections of the manuscript and abstract, and usually of moderate or high degree. We expect researchers use this methodology to avoid or mostly minimize spin in the reports of their NI trials and that this improves the correct and balanced interpretation of their findings.

Our study highlights that reporting of the methodology of NI HIV trials is still deficient in comparison to previous evaluations [2], [51]. Although some time is necessary to adopt recommendations from guidelines, we strongly suggest following the checklist of the CONSORT statement on NI trials [3]. Several journals have adopted the CONSORT guidelines and its extensions, but some major infectious diseases journals are not among the endorsers [52]. If investigators do not appropriately report basic information about the methodology and interpretation, physicians and policy makers may be misled with the conclusions of these trials. However, there is no formal publication evaluating the effects of following the CONSORT guidelines on the reporting of NI trials. This has been done for superiority trials, where following guidelines improved the quality of reporting [4].

### Limitations

There are some limitations to our study. Some specific methodological items might have been conducted but not reported by the authors in the reports we assessed. We did not contact individual trial investigators for any missing items in their reports or trial protocols; instead we solely relied upon what was reported of specific items. There is a degree of subjectivity involved in assessment of spin. However, we pre-specified the evaluation of the presence, extent and degree of spin. We tried to limit this investigator driven bias by performing data extraction to a standardized data extraction sheet. This was done independently by three reviewers and disagreements were resolved by consensus.

### Summary and Recommendations

We described the most comprehensive systematic review to date of NI RCTs in HIV literature. Our findings demonstrate the prevalence of deficiencies in design, reporting and interpretation of NI RCTs in ARV-naïve and ARV-experienced HIV patients. There is a clear need for improving standards of methodology and reporting by following established guidelines when designing and evaluating RCTs. Reviewers of journals, as well as readers should be more aware of these shortcomings in reports of NI RCTs in HIV patients. Rigorous implementation of higher standards in trial design and fully transparent reporting of results will not only improve reliability of the studies but also lead to appropriate appraisal, interpretation and application of results to patient care.

## Supporting Information

Table S1 Study design characteristics stratified by year published.
(DOCX)

Table S2 Study design characteristics stratified by type of sponsor.
(DOCX)

Table S3 Spin in trials where non-inferiority was not established or was inconclusive by year published.
(DOCX)

Table S4 Spin in trials where non-inferiority was not established or was inconclusive by type of sponsor.
(DOCX)

Table S5 PRISMA 2009 checklist.
(DOCX)

Text S1 PubMed search strategy.
(DOCX)
