# Supplementary material for: Deficient Reporting and Interpretation of Non-Inferiority Randomized Clinical Trials in HIV Patients: A Systematic Review
Source: PLoS One. 2013 May 3;8(5):e63272. doi: 10.1371/journal.pone.0063272 (PMC3643946; doi:10.1371/journal.pone.0063272)
Supplement: Table S1 — Study design characteristics stratified by year published. (DOCX) [file pone.0063272.s001.docx]

| **Table S1. Study design characteristics stratified by year published** | | |
| --- | --- | --- |
|  | **Trials published before 2007 (n = 11)** | **Trials published since 2007 (n = 31)** |
| NI margin |  | 7 (1) |
|  |  | 10 (7) |
|  | 10 (2) | 12 (14) |
|  | 12 (5) | 12.5 (1) |
|  | 13 (1) | 14 (1) |
|  | 15 (3) | 15 (3) |
|  |  | 20 (1) |
|  |  | 25 (1) |
|  |  | NA* (2) |
| Method of selection of NI margin |  | Guidelines (2) |
|  | Investigator’s assumption (1) | Investigator’s assumption (1) |
|  | Other publications or reviews (3) | Other publications or reviews (1) |
|  | Investigator’s assumption and other publications or reviews (1) | Calculated by investigator based on previous trials’ results (2) |
|  | Not clear (6) | Guidelines and calculated by investigator based on previous trials’ results (1) |
|  |  | Not clear (24) |
| Sample size calculation used NI margin | No (7) | No (14) |
|  | Yes (4) | Yes (17) |
| 1 or 2 sided confidence intervals | 1-sided (1) | 1-sided (4) |
|  | 2-sided (10) | 2-sided (27) |
| Blinding method | Open label (8) | Open label (25) |
|  | Double blind (3) | Double blind (6) |
| Statistical analysis | Intention-to-treat (7) | Intention-to-treat (12) |
|  | Intention-to-treat and per protocol (3) | Per protocol (2) |
|  | Not clear (1) | Intention-to-treat and per protocol (16) |
|  |  | Not clear (1) |
| Main conclusion based on | Intention-to-treat (10) | Intention-to-treat (21) |
|  | Not clear(1) | Per protocol (4) |
|  |  | Intention-to-treat and per protocol (5) |
|  |  | Not clear (1) |
