# Supplementary material for: Deficient Reporting and Interpretation of Non-Inferiority Randomized Clinical Trials in HIV Patients: A Systematic Review
Source: PLoS One. 2013 May 3;8(5):e63272. doi: 10.1371/journal.pone.0063272 (PMC3643946; doi:10.1371/journal.pone.0063272)
Supplement: Table S2 — Study design characteristics stratified by type of sponsor. (DOCX) [file pone.0063272.s002.docx]

**Table S2. Study design characteristics stratified by type of sponsor**

|  | **Trials sponsored by government (n = 10)** | **Trials sponsored by pharmaceutical companies (n = 27)** |
| --- | --- | --- |
| NI margin | 7 (1) |  |
|  | 10 (3) | 10 (5) |
|  | 12 (2) | 12 (16) |
|  | 15 (1) | 14 (1) |
|  | 20 (1) | 15 (5) |
|  | 25 (1) |  |
|  | NA* (1) |  |
| Method of selection of NI margin |  | Guidelines (2) |
|  |  | Investigator’s assumption (1) |
|  |  | Other publications or reviews (4) |
|  | Calculated by investigator based on previous trials’ results (1) | Investigator’s assumption and other publications or reviews (1) |
|  | Not clear (9) | Guidelines and calculated by investigator based on previous trials’ results (1) |
|  |  | Not clear (18) |
| Sample size calculation used NI margin | No (2) | No (16) |
|  | Yes (8) | Yes (11) |
| 1 or 2 sided confidence intervals | 1-sided (1) | 1-sided (3) |
|  | 2-sided (9) | 2-sided (24) |
| Blinding method | Open label (10) | Open label (18) |
|  |  | Double blind (9) |
| Statistical analysis |  | Intention-to-treat (9) |
|  | Intention-to-treat (7) | Per protocol (2) |
|  | Intention-to-treat and per protocol (3) | Intention-to-treat and per protocol (15) |
|  |  | Not clear (1) |
| Main conclusion based on |  | Intention-to-treat (20) |
|  | Intention-to-treat (8) | Per protocol (3) |
|  | Intention-to-treat and per protocol (2) | Intention-to-treat and per protocol (3) |
|  |  | Not clear (1) |
