# Supplementary material for: Deficient Reporting and Interpretation of Non-Inferiority Randomized Clinical Trials in HIV Patients: A Systematic Review
Source: PLoS One. 2013 May 3;8(5):e63272. doi: 10.1371/journal.pone.0063272 (PMC3643946; doi:10.1371/journal.pone.0063272)
Supplement: Table S3 — Spin in trials where non-inferiority was not established or was inconclusive by year published. (DOCX) [file pone.0063272.s003.docx]

**Table S3. Spin in trials where non-inferiority was not established or was inconclusive by year published**

| **Spin** | **Trials published before 2007 (n = 3)** | **Trials published since 2007 (n = 13)** |
| --- | --- | --- |
| Strategy of Spin |  |  |
| Focus on statistically significant results (within-group comparisons, secondary outcomes, subgroup analysis, modified population of analysis) | 2 | 6 |
| Interpreting the negative results of primary outcome as showing equivalence | 2 | 0 |
| Claiming or emphasizing the non-inferiority despite not-establishing non- inferiority/inconclusive | 3 | 3 |
| Extent of spin in abstract |  |  |
| Results section only | 0 | 0 |
| Conclusions section only | 1 | 3 |
| Results and conclusions sections | 2 | 4 |
| Level of spin in conclusions of the abstract |  |  |
| High spin | 3 | 3 |
| Moderate spin | 0 | 1 |
| Low spin | 0 | 3 |
| Extent of spin in main text |  |  |
| Discussion section only | 1 | 1 |
| Conclusions section only | 0 | 1 |
| Discussion and conclusions sections | 1 | 2 |
| Results and discussion sections | 1 | 2 |
| Results and conclusions sections | 0 | 1 |
| Results, discussion and conclusions sections | 0 | 1 |
| Level of spin in conclusions of the main text |  |  |
| High spin | 1 | 3 |
| Moderate spin | 2 | 1 |
| Low spin | 0 | 4 |
