# Supplementary material for: Deficient Reporting and Interpretation of Non-Inferiority Randomized Clinical Trials in HIV Patients: A Systematic Review
Source: PLoS One. 2013 May 3;8(5):e63272. doi: 10.1371/journal.pone.0063272 (PMC3643946; doi:10.1371/journal.pone.0063272)
Supplement: Text S1 — PubMed search strategy. (DOCX) [file pone.0063272.s006.docx]

**Text S1. PubMed search strategy**

((non[All Fields] AND inferiority[All Fields] AND ("clinical trials as topic"[MeSH Terms] OR ("clinical"[All Fields] AND "trials"[All Fields] AND "topic"[All Fields]) OR "clinical trials as topic"[All Fields] OR "trial"[All Fields])) OR (equivalence[All Fields] AND ("clinical trials as topic"[MeSH Terms] OR ("clinical"[All Fields] AND "trials"[All Fields] AND "topic"[All Fields]) OR "clinical trials as topic"[All Fields] OR "trial"[All Fields])) OR (active-control[All Fields] AND ("clinical trials as topic"[MeSH Terms] OR ("clinical"[All Fields] AND "trials"[All Fields] AND "topic"[All Fields]) OR "clinical trials as topic"[All Fields] OR "trial"[All Fields]))) AND ((antiretroviral[All Fields] AND ("therapy"[Subheading] OR "therapy"[All Fields] OR "therapeutics"[MeSH Terms] OR "therapeutics"[All Fields])) OR ("antiretroviral therapy, highly active"[MeSH Terms] OR ("antiretroviral"[All Fields] AND "therapy"[All Fields] AND "highly"[All Fields] AND "active"[All Fields]) OR "highly active antiretroviral therapy"[All Fields] OR "haart"[All Fields]))
